# A Soil Washing Approach to Remediation of Lead-Contaminated Soil with Amino Acid Ionic Liquid [Met][NO_3_]

**DOI:** 10.3390/toxics13090725

**Published:** 2025-08-28

**Authors:** Yun Deng, Sheng Wang, Lin Fu, Weijie Xue, Changbo Zhang, Jiawei Deng, Xin Luo, Yuyao Liu, Danyang Zhao, Gilles Mailhot

**Affiliations:** 1 School of Environment and Ecology, Jiangnan University, Wuxi 214122, China; dengyun@jiangnan.edu.cn (Y.D.); w1095658753@163.com (S.W.); 2 Key Laboratory of Original Agro-Environmental Pollution Prevention and Control, Agro-Environmental Protection Institute of Ministry of Agriculture and Rural Affairs (MARA), Tianjin 300191, China; zhangchangbo@caas.cn (C.Z.); d17736218037@163.com (J.D.); l_xin01@163.com (X.L.); 15590811177@163.com (Y.L.); taiyang020401@126.com (D.Z.); 3 Institut de Chimie de Clermont-Ferrand, Centre National de la Recherche Scientifique (CNRS), Université Clermont Auvergne, F-63000 Clermont-Ferrand, France; gilles.mailhot@uca.fr

**Keywords:** lead-contaminated soil, soil washing, [Met][NO_3_], soil texture, soil remediation

## Abstract

Against the challenge of extreme lead (Pb) contamination (>15,000 ppm) in industrial polluted soils, where conventional agents fail to disrupt stable Pb–soil complexes—this study extends our prior cadmium (Cd) remediation research to validate amino acid ionic liquids (AAILs) for highly recalcitrant metals. Fifteen AAILs were screened via batch washing, with [Met][NO_3_] (methionine-based) demonstrating the highest Pb removal efficiency. Single-factor optimization revealed that under the conditions of 0.8 mol/L, 6:1 liquid–soil ratio, 60 min, 85.4% Pb was removed from severely contaminated soil by [Met][NO_3_]. Kinetic analysis using four common models showed that the second-order kinetic equation provided the best fit, indicating that Pb removal was predominantly driven by chemical reactions such as complexation or ion exchange. After washing, the contents of various Pb species were significantly reduced, thereby mitigating environmental risks. Notably, no substantial changes in soil texture were observed. However, a marked increase in organic matter content was detected, accompanied by decreases in soil pH and mineral element concentrations. Analysis of soil mineral composition, functional groups, and chemical speciation revealed that [Met][NO_3_] primarily facilitated Pb removal through ion-exchange and coordination reactions. This study establishes [Met][NO_3_] as a green agent with dual efficacy: it achieves high-efficiency remediation of severely Pb-contaminated soil while ensuring environmental sustainability, thus highlighting its potential for practical application.

## 1. Introduction

Soil washing is a high-efficiency remediation technology with short treatment cycles and reliable efficacy, but due to its high cost, its current application is selectively considered in heavily metal-contaminated sites (e.g., industrial zones, mining areas) where conventional methods (e.g., phytoremediation, stabilization) fail due to extreme metal concentrations [1,2]. In some historical smelting zones or uncontrolled waste disposal sites, Pb contamination can reach extreme levels (e.g., >10,000 mg/kg, with reported peaks of 82,752 mg/kg in specific mining tailings), posing significant challenges for conventional remediation approaches. However, critical knowledge gaps persist regarding its scalability, long-term environmental sustainability, and secondary risks (e.g., soil structure degradation, leaching agent residues), particularly for strongly complexed metals like Pb [3].

The washing agent’s composition critically determines removal efficiency, with common agents falling into four categories: inorganic acids (e.g., HNO_3_, HCl, FeCl_3_), organic acids (e.g., citric, oxalic acids), chelators (e.g., EDTA, EDDS) and surfactants (e.g., sodium dodecyl sulfate, saponin and rhamnolipid) [1,4]. However, these agents present significant drawbacks. Inorganic acids can degrade soil structure, reduce fertility, and exacerbate acidification or salinization [5]. Organic acids often exhibit low metal removal efficiency [6], while chelators may persist due to poor biodegradability [7], and surfactants can introduce ecotoxicity [1]. These limitations pose the urgent need for green, high-efficiency alternatives that minimize secondary impacts.

Aligning with “Green Chemistry” principles, Tao et al. [8] developed amino acid ionic liquids (AAILs), which are synthesized from renewable, non-toxic precursors (amino acids + stronger acids) via an energy-efficient, waste-free process. AAILs combine low toxicity, biodegradability, and conventional ionic liquid properties. Our prior works demonstrated AAILs’ efficacy in Cd-contaminated farmland [9] and As-contaminated industrial soil [10], achieving high metal removal without soil function damage. Encouragingly, the soil type remained unaltered and the content of nutrient elements increased, but the soil pH and mineral content decreased. The potential mechanisms of heavy metals removal by AAILs included acid dissolution, ion exchange and coordination reactions. Furthermore, given that the amino acid ions are with biocompatibility and have no risk of secondary pollution, the washing waste solution can be recycled after simple treatment. For instance, by adopting the method of “targeted removal of adsorbates to retain functional components” [11], after removing heavy metal ions from the waste solution, the supernatant rich in free AAILs can be reused in the leaching process after supplementing an appropriate amount of AAILs to the initial concentration. However, extreme Pb contamination in industrial soils poses a greater challenge due to Pb’s strong binding with soil matrices, rendering conventional agents ineffective.

Notably, a critical research gap remains: extreme Pb contamination in industrial soils, characterized by Pb’s strong binding with soil matrices (far stronger than that of Cd or As), has proven recalcitrant to conventional agents. Pb exhibits distinct geochemical behavior in soil compared to other toxic metals: it has a far stronger affinity for organic matter and iron/manganese oxides, with a larger portion bound to Fe/Mn oxides than Cd or As [12]. Moreover, Pb tends to form highly insoluble phosphates and carbonates—reacting with soil calcium carbonate to generate insoluble lead carbonate, and with available phosphorus to form extremely stable compounds like Pb-phosphate and pyromorphite—effectively fixing Pb in soil and reducing its mobility [13]. These unique characteristics make extreme Pb contamination a largely unaddressed challenge, as existing remediation strategies (including AAILs applied to Cd and As) lack validation under such harsh conditions.

Building on the previous success, this study addresses the unmet challenge of extreme Pb contamination (16,654 mg/kg). The objective was to validate AAILs as a green alternative for Pb removal under severe contamination, while minimizing soil function disruption. This research represents a novel extension of AAILs’ applicability to highly recalcitrant metals, establishing a dual-efficacy framework for extreme Pb contamination: efficient Pb desorption via combined acidity and chelation, paired with preservation of soil microbial activity and nutrient balance—advancing beyond our prior Cd studies by demonstrating efficacy under far more demanding conditions.

## 2. Materials and Methods

### 2.1. Preparation of Pb-Contaminated Soil

The test soil was obtained from the surface layer (0–20 cm) in the vicinity of the pond located at the School of Environment and Ecology at Jiangnan University, Wuxi, Jiangsu Province. The sand/Silt/Clay (%), pH, organic matter (OM) content, electrical conductivity (EC) and cation exchange capacity (CEC) was 35/49/16, 6.4, 7.6 g/kg, 0.94 ms/cm and 15.5 cmol/kg. The soil sample was first subjected to impurity removal and air drying, after which it was ground and sifted through a 10-mesh screen. Subsequently, the resulting soil was thoroughly blended with a Pb(NO_3_)_2_ solution and stirred periodically for two weeks, with the soil moisture content maintained at approximately 60%. After aging at room temperature for six months, the soil samples were naturally air-dried, reground, and sieved again through a 10-mesh screen. Classified as loam based on particle size distribution, the contaminated soil had a Pb concentration of 16,654 ± 354.8 mg/kg.

### 2.2. Preparation of Washing Agents

**Figure 1:**
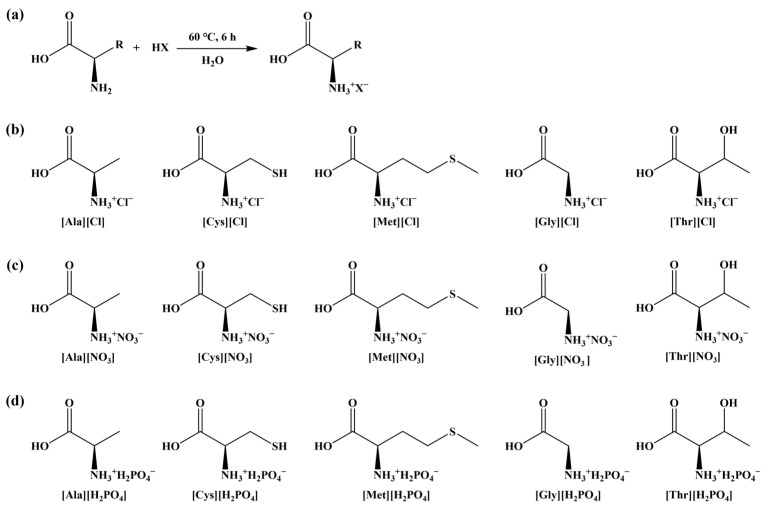
Synthesis scheme (**a**) and structural formula (**b**–**d**) of AAILs. R represents the side chain groups of amino acids, and HX represents the inorganic acids.

In this study, following the procedure described by Tao et al. [8], 15 AAILs were synthesized using five amino acids (alanine (Ala), cysteine (Cys), methionine (Met), glycine (Gly), threonine (Thr)) and three inorganic acids (hydrochloric acid (HCl), nitric acid (HNO_3_), phosphoric acid (H_3_PO_4_)). All the chemicals used were analytically pure. An equal molar mass of amino acids and inorganic acids were thoroughly mixed in deionized water. The mixed solution was stirred at 60 °C for 6 h to obtain AAILs, which are denoted as [AA][X] (where [AA] represents amino acids ion and [X] represents acid ions). The synthesis scheme and structural formula are shown in Figure 1.

### 2.3. Design of Soil Washing Experiment

Batch washing experiments were conducted to identify the AAILs with the highest removal efficiency of Pb from contaminated soil. Subsequently, the most efficient washing agent was selected for further investigation of the effect of washing agent concentration, liquid-to-soil ratio, and washing time on the removal efficiency of Pb by single-factor experiments. In the washing experiment, 5.0 g of the soil sample was placed into a 50 mL centrifuge tube, and a specific volume of AAILs solution was then added in accordance with the experimental objective. The mixture was then oscillated at 150 r/min for a predetermined time. Afterward, the suspension was centrifuged at 4000 r/min for 10 min, after which the supernatant was filtered with 0.45 μm filtration membrane to obtain the solution to be detected. All experiments conducted in this study were repeated on three occasions, and deionized water (DI water) from an ultra-pure water machine (EPED-20TH, Shanghai, China) was employed as the washing agent in the control group. The detailed experimental parameters are shown in Table 1. After washing, some samples were directly air-dried without water rinsing for the determination of physicochemical properties; other samples were freeze-dried at −80 °C and then used for scanning electron microscopy (SEM), X-ray diffraction (XRD), Fourier-transform infrared spectroscopy (FTIR) and X-ray electron spectrometer (XPS).

All experimental data are expressed as “mean ± standard deviation” (*n* = 3). One-way analysis of variance (One-way ANOVA) was used to test the significance of differences among different treatment groups. In the preliminary tests, the Shapiro–Wilk test was employed to verify the normality of the data, and the Levene test was used to check the homogeneity of variances. Based on a significant result from the analysis of variance (*p* < 0.05), Duncan’s multiple comparison method was applied for pairwise comparisons between groups. 

### 2.4. Detection Method

#### 2.4.1. Determination of Pb Content and Pb Form

HNO_3_-HCl-HF was used for the digestion of soil samples, which occurred on a microwave digestion instrument (SHB-III, AntonPaar, Graz, Austria). The heating procedure of the microwave digester comprised three distinct stages. In the initial stage, the temperature increased from the ambient temperature to 120 °C, with a heating time of 10 min followed by a digestion time of 5 min. The second stage involved raising the temperature to 160 °C, with a heating time of 3 min and a digestion time of 5 min. In the third stage, the temperature was increased to 185 °C, with a heating time of 10 min and a digestion time of 20 min. After digestion, the digestion solution was transferred to a 50 mL polytetrafluoroethylene crucible, which was placed on an electric furnace at 200 °C to remove the acid. Subsequently, the solution was filled to 50 mL with DI water and filtered through a 0.45 μm filter membrane, thereby obtaining the Pb detection solution. Tessier five-step extraction method was used to extract different forms of Pb in soil samples, including exchangeable Pb (F1), carbonate-bound Pb (F2), iron–manganese-oxide-bound Pb (F3), organic-bound Pb (F4), and residual Pb (F5). Table 2 shows the detailed operations.

The concentration of Pb in soil samples and washing waste liquid was determined by a flame atomic absorption spectrophotometer (AA-7000, Shimadzu, Kyoto, Japan). A lead hollow cathode lamp was selected as the light source for the instrument, and the flame type was set to a neutral flame. The specific parameters were set as follows: the lamp current was 8.0 mA, the measurement wavelength was 283.3 nm, and the spectral bandwidth is 0.5 nm.

#### 2.4.2. Determination of Physical and Chemical Properties of Soil

The basic physical and chemical properties (particle size distribution, pH, electrical conductivity (EC), organic matter (OM)), soil exchange properties (cation exchange capacity (CEC), exchangeable calcium (Ex-Ca), exchangeable magnesium (Ex-Mg)), soil nutrients content (total nitrogen (TN), total phosphorus (TP), total potassium (TK), available phosphorus (AP), available potassium (AK)), and predominant mineral elements content (calcium (Ca), magnesium (Mg), manganese (Mn), iron (Fe)) of the soil samples were determined, as shown in Table 3.

#### 2.4.3. Characterization of Soil

The soil samples were characterized to analyze surface morphology, mineral composition, functional groups, and chemical morphology, as shown in Table 4.

### 2.5. Data Analysis

The removal efficiency of Pb (*R*%) was expressed by the proportion of Pb removal to the total amount, and the calculation formula was as follows:

(1) $$R\left(\%\right)=\frac{C\cdot V}{w\cdot m},$$

where *C* (mg/kg) represents the concentration of Pb in the washing waste liquid; *V* (L) represents the volume of the washing waste liquid; *w* (mg/kg) represents the concentration in the soil before washing; *m* (kg) represents the mass of the soil particles. Since the supernatant was passed through a 0.45 μm aqueous filter membrane, which served to remove any residual fine soil particles and minimize the loss of soil particles from the solid phase, the mass of soil particles carried in the filtrate accounted for less than 0.5% of the initial soil mass, indicating that the loss was negligible.

The kinetic model was used to investigate the dynamic characteristics of Pb removal in soil, and the equation is shown in Table 5. Statistical analysis and model development were performed using the Nonlinear Curve Fitting module of Origin 2023 software. Based on the nonlinear expressions of each model, fitting was conducted by minimizing the sum of squared residuals between measured and predicted values via the least square method. The Levenberg–Marquardt algorithm was applied to optimize parameters during fitting.

All experiments were conducted in triplicate, and the resulting data were expressed as mean ± standard deviation. The relative deviation of the parallel samples was within the allowable range.

## 3. Results and Discussion

### 3.1. Effect of Different AAILs on Pb Removal from Soil

**Figure 2:**
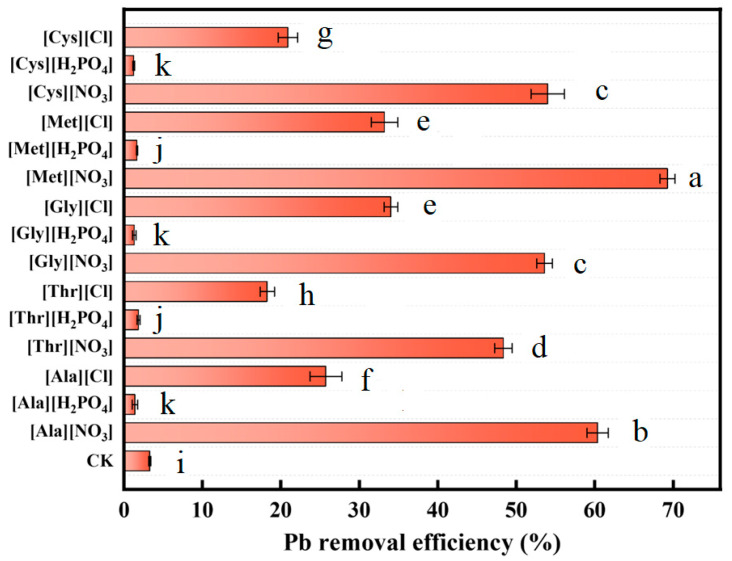
Effect of different AAILs on removal efficiency of Pb from soil. The letters indicate the significance grouping marking (α = 0.05), where the same letter indicates no significant difference between groups, and different letters indicate significant differences.

The removal efficiency of Pb in soil by washing of different AAILs is shown in Figure 2. In the control group, a mere 3.3% of Pb was removed from the soil after washing with DI water. Among the 15 AAILs tested, [Met][NO_3_] exhibited the highest Pb removal efficiency at 69.3%, followed by [Ala][NO_3_] with a removal efficiency of 60.4%. Conversely, the removal efficiency of Pb by [Cys][H_2_PO_4_] was the lowest, only 1.3%. Statistical analysis via one-way ANOVA followed by Duncan’s multiple comparison test revealed significant differences among the groups (*p* < 0.05), with [Met][NO_3_] showing a significantly higher removal efficiency than all other treatments, and [Cys][H_2_PO_4_] being significantly lower than the control group. It is noteworthy that the Pb removal efficiency of AAILs with H_2_PO_4_^−^ as the anion was relatively low, ranging from 1.3% to 1.9%, which was even inferior to that of the control group. This may be due to the lower solubility of Pb^2+^ compounds with phosphate in soil, in comparison to other lead-containing compounds [14]. Furthermore, AAILs with NO_3_^−^ anion exhibited higher washing efficiency those employing Cl^−^ and H_2_PO_4_^−^ as anions. Such trends were statistically validated, with NO_3_^−^-based AAILs forming a distinct significant group compared to Cl^−^ and H_2_PO_4_^−^-based ones. This discrepancy may be attributed to the propensity of Cl^−^ and H_2_PO_4_^−^ to precipitate with Pb^2+^, thereby limiting the removal [15]. Among the NO_3_^−^-based AAILs, [Met][NO_3_] demonstrated best performance, which may be due to the sulfur-containing groups on the side chain of Met that can form ligands with Pb^2+^ [16]. In comparison to reported inorganic washing agents (31.5–39.6%), chelators (1.1–63.0%), and organic acids (1.1–60.0%), [Met][NO_3_] has superior efficacy in the removal of Pb from the soil [17].

Although the current price of AAILs is higher than that of the conventional chelators, such as EDTA and citric acid, EDTA is difficult to biodegrade and tends to accumulate in the environment, resulting in long-term pollution, while the chelating efficiency of citric acid is significantly affected by pH value, increasing operational difficulties in complex soil environments and limits its application scope. Nevertheless, if amino acid production wastewater can be used as a raw material source, the preparation cost of AAILs will be greatly reduced, creating conditions for their large-scale application. This enables them to have cost advantages while taking into account environmental benefits, making them a highly potential alternative to traditional eluents. On this basis, [Met][NO_3_] is selected as the target for further investigation in the following sections.

### 3.2. Effect of Different Washing Conditions on Pb Removal from Soil

#### 3.2.1. Concentration

**Figure 3:**
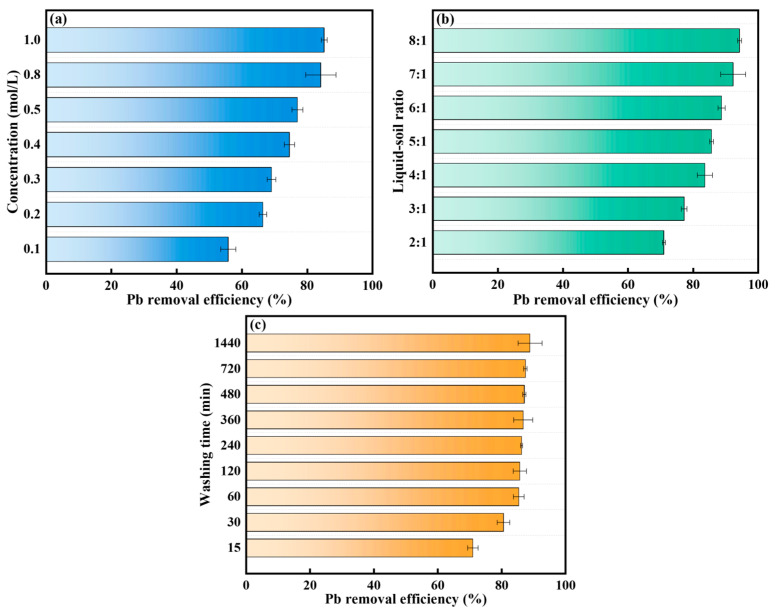
The effect of concentration (**a**), liquid–soil ratio (**b**) and washing time (**c**) on the removal efficiency of Pb from soil.

The removal efficiency of Pb in the soil varies with changes in the concentration of [Met][NO_3_] (Figure 3a). An increase in the concentration of [Met][NO_3_] from 0.1 mol/L to 0.8 mol/L was observed to cause a significant enhancement in the removal efficiency of Pb, with a corresponding increase from 55.8% to 84.2%. Nevertheless, when the concentration of [Met][NO_3_] exceeded 0.8 mol/L, there was minimal variation in the removal efficiency of Pb. At a concentration of 1.0 mol/L, the removal efficiency of Pb in soil was 85.2%, indicating that 0.2 mol/L increase in the washing agent concentration resulted in only a 1.0% improvement in the removal efficiency of Pb. The results indicated that within a specific concentration range (0–0.8 mol/L), a higher dosage of [Met][NO_3_] was more effective in removing Pb from the soil. This may be due to the fact that an increase in the concentration of [Met][NO_3_] resulted in an increase in the functional groups that interact with Pb^2+^, which facilitated the coordination reaction and subsequently enhanced the efficiency of the removal efficiency of Pb by washing [18,19]. Beyond this range (>0.8 mol/L), the removal efficiency of Pb appeared to reach a state of equilibrium. Additionally, the co-solubility effect between Pb and co-existing metals (such as Ca, Mg, Fe, and Al) may also lead to reduced removal efficiency, especially at lower concentrations of the washing agent [20,21]. Considering the financial and environmental advantages associated with soil remediation, a concentration of 0.8 mol/L was identified as the optimal level for washing Pb-contaminated soil with [Met][NO_3_]. Increasing the concentration beyond 0.8 mol/L not only causes a sharp rise in material costs and exacerbates adverse effects on the soil, but also results in only a negligible improvement in removal efficiency, making it clearly not worth the cost.

#### 3.2.2. Liquid–Soil Ratio

The liquid–soil ratio also affects the removal efficiency of Pb in the washing process, as shown in Figure 3b. An increase in the liquid–soil ratio from 2:1 to 6:1 has been observed to result in a notable enhancement in the removal efficiency of Pb by [Met][NO_3_], with an increase from 71.0% to 88.7%. Upon further increasing the liquid-to-soil ratio from 6:1 to 8:1, the removal efficiency of Pb by [Met][NO_3_] increased from 88.7% to 94.2%, while the rate of increase diminished. A lower liquid–soil ratio resulted in a reduced washing agent volume, which in turn led to an uneven distribution of soil and detergent, resulting in a pulpy reaction system [22]. Consequently, the removal efficiency of Pb from the soil was inhibited. In contrast, a higher liquid–soil ratio allowed for an increased number of functional groups that can participate in the reaction, thereby improving the removal efficiency of Pb [23,24]. Furthermore, an elevated liquid–soil ratio facilitates optimal contact between the soil and the washing agent [25]. Nevertheless, a high liquid–soil ratio requires greater energy expenditure, which not only complicates the washing process but also produces a greater volume of waste liquid that must be managed [18]. It is also crucial to consider the potential negative impacts of high liquid–soil ratios on soil properties. To balance Pb removal efficiency with soil fertility preservation and acidification control, a comprehensive assessment was conducted: although 8:1 achieved the highest Pb removal (94.2%), it caused more severe nutrient loss and acidification; 4:1 mitigated these risks but resulted in a relatively low removal efficiency (71.0%). In comparison, 6:1 maintained a high Pb removal efficiency (88.7%) with manageable nutrient leaching and acidification, which can be partially alleviated by subsequent measures such as adding slow-release alkalis (e.g., calcium carbonate) and nutrient supplements if needed. Therefore, a liquid–soil ratio of 6:1 was selected for the washing of Pb-contaminated soil in the present study.

#### 3.2.3. Washing Time

For the removal of Pb from the soil, the washing time is an important factor affecting its efficiency. The removal efficiency of Pb using [Met][NO_3_] washing with washing time is shown in Figure 3c. In the initial phase of the washing process (0–60 min), the removal efficiency of Pb increased rapidly with extended washing time. When the washing time reached 60 min, the removal efficiency of Pb was 85.4%. In the late stage of the washing process (60–1440 min), the removal efficiency of Pb increased more gradually, from 85.4% to 88.8%. Furthermore, when the washing time exceeded 240 min, the removal efficiency of Pb tended to approach an equilibrium. These results indicated that Pb removal from the soil by washing with [Met][NO_3_] comprised two stages: a rapid initial stage within the first 60 min, followed by a slower increase in efficiency as it reached a state of equilibrium. This phenomenon was consistent with findings from other studies investigating the influence of washing time on the removal efficiency of heavy metals [1,26,27]. Physically, the rapid initial stage (0–60 min) primarily corresponds to the desorption of “unstable Pb” from soil surface regions and large pores. These Pb fractions are weakly bound to soil particles (e.g., adsorbed on mineral surfaces via electrostatic forces or complexed with easily degradable organic matter), and [Met][NO_3_] can quickly access these sites through mass transfer in the liquid phase. The high mobility of the washing agent in large pores facilitates efficient interaction with Pb, leading to rapid desorption and dissolution. In contrast, the slow stage (60–1440 min) is dominated by the release of “strongly bound Pb,” which is trapped in soil micropores or incorporated into the crystal lattice of minerals (e.g., substituted in phyllosilicate structures or encapsulated in iron/manganese oxides). Diffusion of [Met][NO_3_] into micropores is hindered by narrow pore throats, resulting in a slower mass transfer rate. Additionally, breaking the crystal lattice to release Pb requires more energy and time, as it involves disrupting chemical bonds within the mineral structure. This dual mechanism explains why the removal rate decelerates significantly after 60 min, as the easily accessible Pb is exhausted and the remaining fraction is more resistant to extraction. The rapid removal of unstable Pb in the initial stage of washing may be the primary factor influencing the removal efficiency, which is subsequently affected by the presence of more firmly bound Pb over time [28]. Following a comprehensive evaluation, the optimal washing time for [Met][NO_3_] washing of Pb-contaminated soil was determined to be 60 min. Prolonging the washing time not only increases energy consumption and operational costs, but also exacerbates adverse effects on the soil. However, the marginal improvement in Pb removal efficiency after 60 min has been negligible, making it not worth extending further.

It should be noticed that, in these single-factor experiments, the results of the previous test were directly applied to the next one. Although this approach enabled efficient screening of optimal conditions for individual factors, it failed to evaluate the interactions between various factors. Future studies could adopt response surface methodology or orthogonal experimental design to quantify the interaction effects among variables through multi-factor and multi-level combinatorial experiments.

#### 3.2.4. Kinetics of Pb Removal

**Figure 4:**
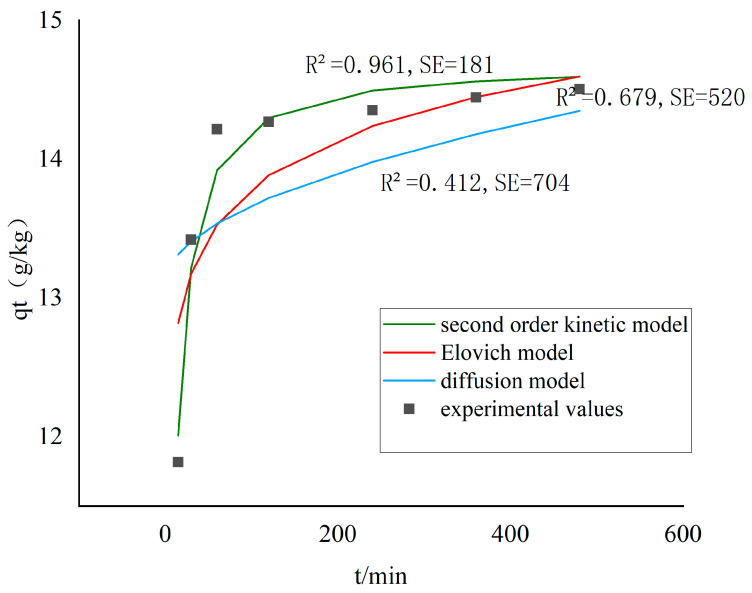
Kinetic fitting curve of Pb removal during washing process. The [Met][NO_3_] washing agent was selected with 0.8 mol/L concentration, 60 min washing time and 6:1 liquid–soil ratio.

To examine the kinetic characteristics of Pb removal in the washing process, three common kinetic models were introduced for fitting the experimental results, as shown in Figure 4. A comparative analysis of the three kinetic models, based on the coefficient of determination (*R*^2^) and standard error (*SE*), revealed that a second-order kinetic equation exhibited the highest value *R*^2^ (0.961) and the lowest *SE* (181), demonstrating superior fitting performance. These results suggested that the removal of Pb by [Met][NO_3_] from soil during the washing process was predominantly driven by chemical reactions, such as complexation reactions or ion exchange. This observation was in accordance with the findings reported in the extant literature [29]. The amino acid ion part in [Met][NO_3_] might change the interfacial properties between soil particles and lead ions, promoting the desorption and removal of lead ions [30].

### 3.3. Effect of Soil Washing on Soil Properties

#### 3.3.1. Chemical Forms of Pb in Soil

**Figure 5:**
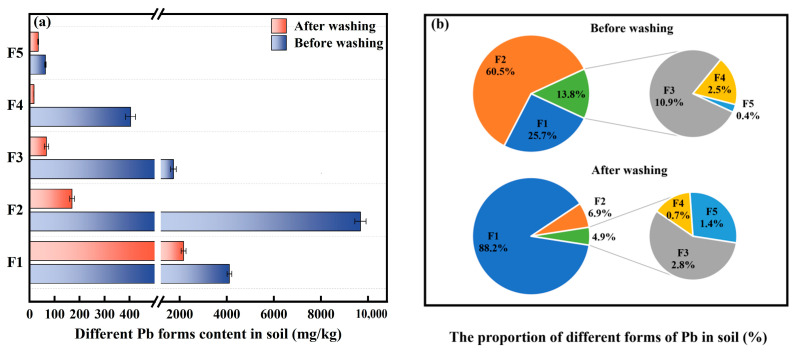
Effect of soil washing on the content (**a**) and proportion (**b**) of Pb forms in soil. The [Met][NO_3_] washing agent was selected with 0.8 mol/L concentration, 60 min washing time and 6:1 liquid–soil ratio. F1 represents exchangeable Pb, F2 represents carbonate-bound Pb, F3 represents iron–manganese-oxide-bound Pb, F4 represents organic-bound Pb, F5 represents residual Pb.

Figure 5 shows the content and distribution of Pb forms in soil before and after washing with [Met][NO_3_]. In the soil before washing, the carbonate-bound Pb was the predominant form, with a concentration of 9679.6 mg/kg, which constituted 60.5% of the total Pb. The content of exchangeable Pb and Fe-Mn-oxide-bound Pb was 4114.9 mg/kg and 1736.4 mg/kg, respectively, accounting for 25.7% and 10.9%, respectively. In contrast, the proportion of organic-bound Pb and residual Pb was relatively minor, amounting to only 2.5% and 0.4%, respectively. In particular, the low concentration of residual Pb may be attributed to the shorter aging time of the simulated heavy metal-contaminated soil.

After washing, a notable reduction in the concentration of all forms of Pb was observed in the soil. To be specific, exchangeable Pb, carbonate-bound Pb, Fe-Mn-oxide-bound Pb, organic-bound Pb, and residual Pb were removed by 47.3%, 98.2%, 96.1%, 95.6%, and 45.3%, respectively. The results indicated that carbonate-bound Pb, Fe-Mn-oxide-bound Pb, and organic-bound Pb were more easily removed by washing with [Met][NO_3_]. This may be attributed to the potential for H^+^ in the washing agent to interact with minerals or functional groups on the soil surface, thereby facilitating the desorption of Pb^2+^ from the soil [31]. Moreover, the residual Pb is predominantly situated within the crystalline structure of the mineral and is regarded as the most stable component. Consequently, its removal represents a significant challenge [32].

#### 3.3.2. Physico-Chemical Properties of Soil

**Figure 6:**
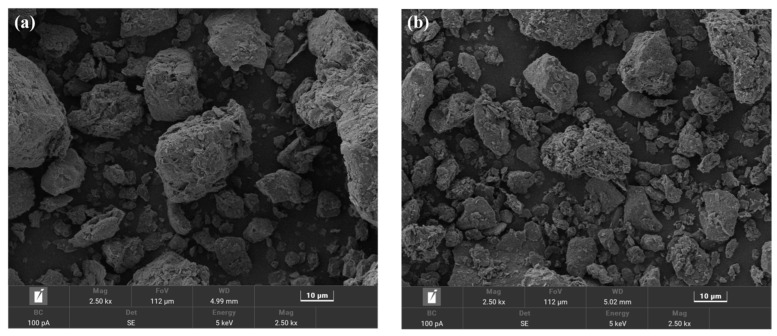
SEM images of soil before (**a**) and after washing (**b**). The [Met][NO_3_] washing agent was selected with 0.8 mol/L concentration, 60 min washing time and 6:1 liquid–soil ratio.

The effect of washing on soil structure was investigated by SEM (Figure 6). After washing, the soil particles were broken down into smaller sizes, and the surface was coarser. This alteration may be due to hydraulic erosion occurring during the washing process, as well as the dissolution of soluble substances present on the soil surface [33].

The soil before washing was composed of sand (36%), silt (47%), and clay (17%) (Table 6), which was classified as loamy soil. After washing with [Met][NO_3_], the proportion of clay in the soil decreased to 15%, while the proportion of sand increased to 38%. However, the alteration was not obvious, indicating that the soil texture did not change fundamentally. This minor fluctuation in sand and clay proportions aligns with the macro-scale stability reflected in Table 6, further confirming that [Met][NO_3_] leaching does not trigger drastic shifts in the overall soil texture classification. The subtle decrease in clay content might be attributed to the detachment of fine clay particles weakly bound to larger aggregates during leaching, yet such changes are insufficient to alter the loamy soil category. Notably, this macro-scale stability contrasts with the microstructural changes observed via SEM (Figure 6). While the sand–silt–clay framework remains intact, the “roughened surface” of soil particles—possibly resulting from the removal of surface coatings like ferromanganese oxides and organic films—reveals finer-scale modifications. These microscopic alterations, though not captured by traditional granulometric analysis, could enhance soil porosity and surface reactivity. For instance, the exposed primary textures of particles may improve water-holding capacity or nutrient retention, which are critical for post-remediation soil functionality. Thus, the combination of minimal macro-texture changes and distinct microstructural adjustments underscores that [Met][NO_3_] leaching achieves effective Pb removal while preserving soil’s basic physical framework, with micro-level optimization potentially supporting ecological recovery.

The pH value of the soil after washing was 2.0, significantly lower than that of the original soil (pH = 5.2) (Table 6). The soil acidification after washing may be caused by residual washing agents in the soil. Therefore, an appropriate amount of alkaline substances can be added after the washing process to alleviate soil acidification. It has been proven that after the soil was rinsed twice with water and the pH was adjusted to 6.2 by adding Ca(OH)_2_, the germination index of the rice increased by 7.5%. The growth of the rice was also stimulated, with lengths and weights of the rice plants increasing by 56% and 32%, respectively, after two weeks [9,10]. EC reflects the content of base ions in the soil. After washing with [Met][NO_3_], the EC increased from 1.9 ms/cm to 4.5 ms/cm (Table 6), likely due to the ionic nature of the washing agent remaining in the soil, leading to an increase in salt content. In order to prevent soil salinization, it is recommended to reduce the soil salt content by rinsing it with water several times after the washing process. In addition, OM is considered to be a crucial indicator of soil fertility, and it also affects the bioavailability and migration capacity of heavy metals in soil [34]. Following the process of washing with [Met][NO_3_], the content of OM in the soil significantly increased from 7.2 g/kg to 23.0 g/kg, more than doubling. This is attributed to the presence of organic carbon in [Met][NO_3_], which is retained in the soil during the washing process. Furthermore, the CEC in the soil increased from 18.4 cmol/kg to 19.4 cmol/kg, with an increase of 5.4%. While the Ex-Ca content decreased from 4.5 g/kg to 1.8 g/kg by 60.0%, and the Ex-Mg content decreased from 2.3 g/kg to 2.0 g/kg by 13.0%. This may be attributed to the fact that the exchangeable form of metal cations in the soil is more readily replaced by H^+^ in the washing agent [35].

#### 3.3.3. Chemical Compositions of Soil

**Figure 7:**
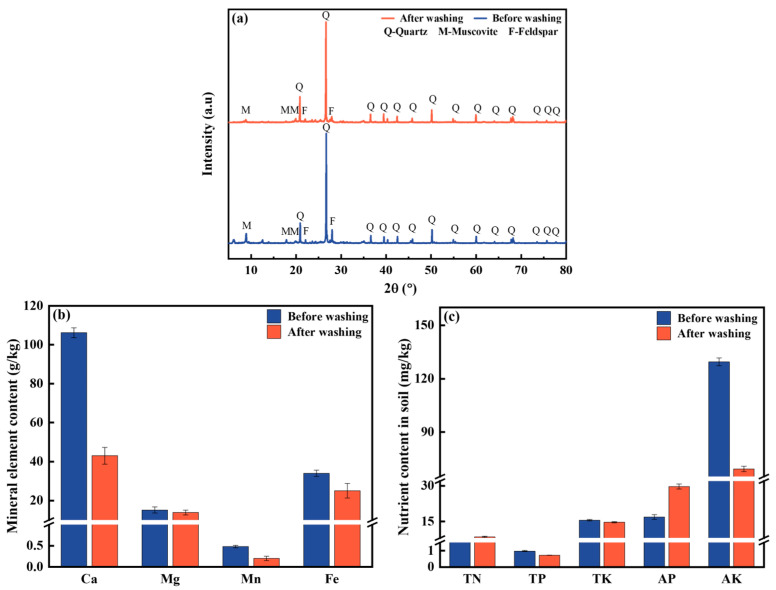
XRD spectra of soil before and after washing (**a**), and the effect of soil washing on mineral element content (**b**) and nutrient content (**c**) in soil. The [Met][NO_3_] washing agent was selected with 0.8 mol/L concentration, 60 min washing time and 6:1 liquid–soil ratio.

The crystal mineral composition of the soil before and after washing was analyzed by XRD, as shown in Figure 7a. The primary components of the soil before washing included quartz (26.70°, 20.92°, 39.53°, 50.19°, 60.02°), muscovite (8.93°, 17.85°, 19.87°), and feldspar (22.11°, 28.01°) [36]. After washing, the positions and intensities of the characteristic peaks of quartz and feldspar demonstrated no discernible trend of change, indicating that these two crystal minerals in the soil were basically unchanged [37]. While the peaks of muscovite decreased, suggesting that the muscovite was partially dissolved [38].

The content of major mineral elements in the soil is shown in Figure 7b. The content of Ca, Mg, Mn, and Fe in soil decreased by 59.5%, 8.5%, 58.3%, and 26.3%, respectively, after washing with [Met][NO_3_]. The loss of these mineral elements may be caused by the dissolution of soil components containing these elements [39], the coordination of the washing agent with the metal ions [20], and/or the replacement of these ions by H^+^ in the washing solution [35].

The content of nutrient elements (N, P, K) in the soil was also tested (Figure 7c). TN content increased significantly, from 1.5 g/kg to 8.4 g/kg, which represented an increase of more than four times. This may be due to the presence of a substantial number of amino and NO_3_^−^ in the AAILs. In contrast, TP content decreased from 1.0 g/kg to 0.7 g/kg, and TK content decreased from 15.5 g/kg to 14.6 g/kg. This decline may be attributed to the acidic nature of [Met][NO_3_], which had the potential to dissolve certain phosphate minerals and insoluble potassium (such as muscovite) in the soil [40,41]. In addition, the content of AP increased significantly from 16.9 mg/kg to 29.7 mg/kg, possibly due to the conversion of TP [42]. Conversely, the content of AK decreased significantly from 129.4 mg/kg to 69.3 mg/kg, possibly because exchangeable potassium was more readily extracted by the washing agent [43].

#### 3.3.4. Functional Groups and Elements Chemical Form of Soil

**Figure 8:**
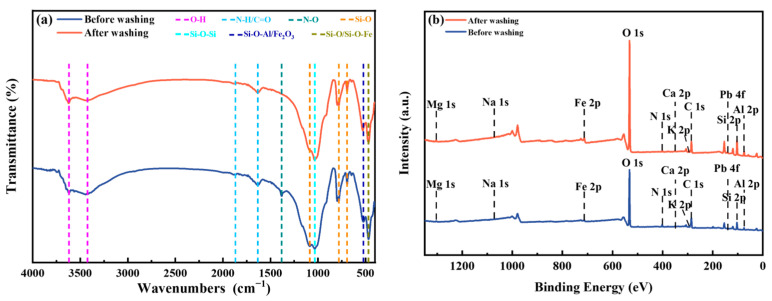
FT-IR spectra (**a**) and Wide-scan XPS spectra (**b**) of soil before and after washing. The [Met][NO_3_] washing agent was selected with 0.8 mol/L concentration, 60 min washing time and 6:1 liquid–soil ratio.

To reveal the potential mechanism of Pb removal by washing, the effect of soil washing on the functional groups in soil was analyzed. The FT-IR spectra of the soil before and after washing are shown in Figure 8a. The absorption peaks of soil samples were mainly concentrated within the range of 3750 cm^−1^ to 3250 cm^−1^, and 2000 cm^−1^ to 400 cm^−1^. The absorption peaks at 3618 cm^−1^ and 3423 cm^−1^ may be due to the stretching vibration of O-H, primarily resulting from the adsorption of water, hydroxyl, and carboxyl groups [44]. The absorption peaks observed from 1888 cm^−1^ to 1633 cm^−1^ were attributed to the vibrations of N-H and C=O in carboxyl and carbonyl groups. Due to the addition of nitrate during the preparation of Pb-contaminated soil, the N-O vibration peak was observed near 1381 cm^−1^ [45]. The intensity of this peak was found to decrease after washing, indicating a reduction in the concentration of NO_3_^−^. Additionally, the absorption peaks within the range of 1085 cm^−1^ to 469 cm^−1^ were associated with the presence of Si-O in quartz, Si-O/Si-O-Fe in quartz or feldspar, Si-O-Si in muscovite, and Si-O-Al/Si-O in quartz or muscovite [46,47]. Notably, the observed absorption peaks remained largely unaltered following washing, which was in accordance with the results of the XRD analysis.

XPS was performed to ascertain the chemical state of surface elements in the soil, as shown in Figure 8b. The analysis of the soil samples revealed the presence of C 1s, O 1s, N 1s, Mg 1s, Na 1s, Fe 2p, K 2p, Ca 2p, Al 2p, and Si 2p, both before washing and after washing, indicating that the main elements of soil composition remain unaltered [48].

**Figure 9:**
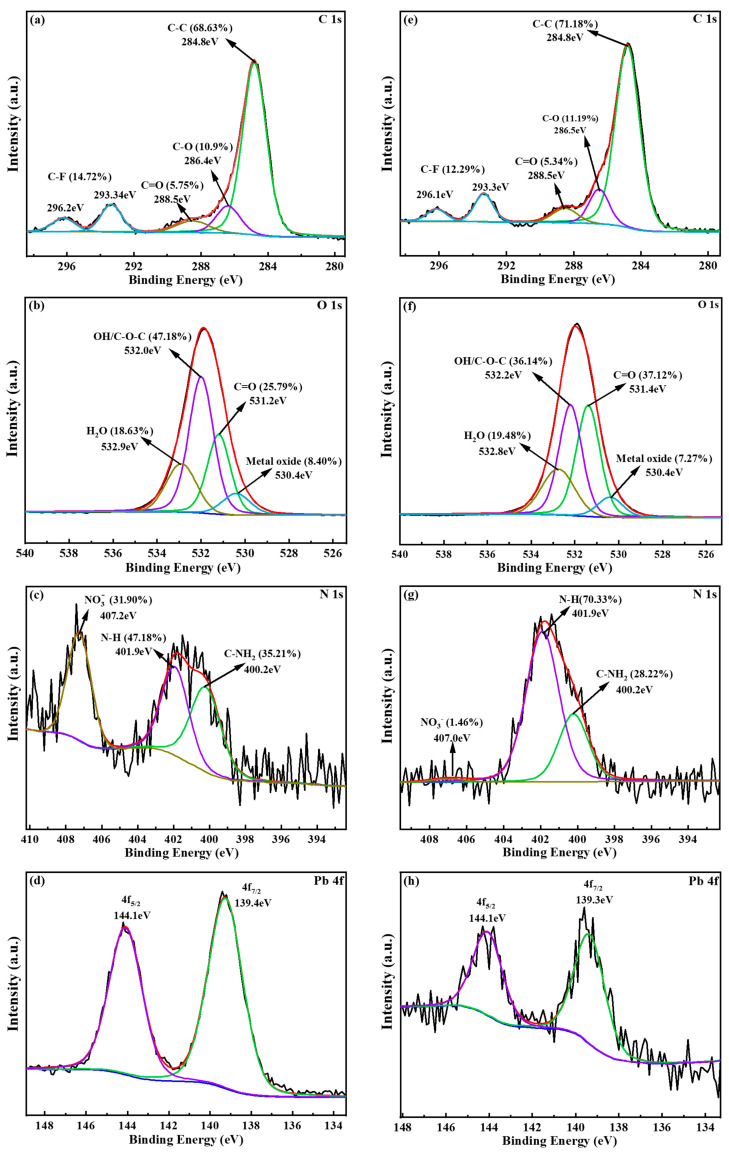
High–resolution XPS spectra of the C 1s, O 1s, N 1s, and Pb 4f in soil before (**a**–**d**) and after washing (**e**–**h**). The [Met][NO_3_] washing agent was selected with 0.8 mol/L concentration, 60 min washing time and 6:1 liquid–soil ratio.

Figure 9 shows the high-resolution XPS spectra of the C 1s, O 1s, N 1s, and Pb 4f in the soil. The C 1s spectrum demonstrated the presence of C-C, C-O, C=O, and C-F [34]. Notably, the content of C-C and C-O in the soil increased after washing, likely due to the high content of C-C and C-O in [Met][NO_3_] remaining on the soil surface. Similarly, the O 1s spectrum exhibited four distinct peaks, which can be attributed to the following chemical species: OH/C-O-C, C=O, adsorbed H_2_O, and metal oxide [49]. The binding energy of C-O and -OH in the soil increased after washing, indicating that these may be involved in the coordination reaction with Pb. Concurrently, considerable variations were observed in the content of C=O and -OH, suggesting their participation in the reactions [50]. Furthermore, the proportion of metal oxide content in the soil was noted to decline after washing, which may be the result of acid dissolution. The N 1s spectrum showed the presence of N-H, C-NH_2_, and NO_3_^−^ in soil [51]. The residue of [Met][NO_3_] in the washing process may result in an increase in the N-H content from 41.8% to 70.3%. Conversely, a reduction in the C-NH_2_ content from 35.2% to 28.2% may be attributed to a reaction between the -NH_2_ group and the metal. Furthermore, the alteration in NO_3_^−^ content was in accordance with those observed through FT-IR analysis. The Pb 4f spectrum displayed two fitting peaks, Pb 4f_7/2_ (139.3~139.4 eV) and Pb 4f_5/2_ (144.1eV), indicating the presence of Pb (II) in the soil. Based on the preceding analysis of Pb forms in soil, it is postulated that the peak of Pb 4f_5/2_ at 144.1 eV was indicative of Pb (II) in PbCO_3_ [52]. Furthermore, the binding energy of Pb 4f in the soil decreased after washing. It is possibly because the lone pair electrons of -COOH and -NH_2_ were biased towards the metal orbital, indicating the coordination with heavy metals [53].

Based on the above results, the primary mechanisms for Pb removal by washing with [Met][NO_3_] may involve ion exchange and coordination. XRD and FT-IR analyses revealed no significant changes in the main functional groups or mineral compositions of Pb-contaminated soil before and after washing, indicating that the removal ability of [Met][NO_3_] for Pb existing in minerals (mainly residual Pb fractions) was limited. Furthermore, XPS characterization confirmed the participation of functional groups such as -OH, -NH_2_, and -COOH in coordinating with Pb during the [Met][NO_3_] washing process.

Given that [Met] is an amino acid ion with biocompatibility and no risk of secondary pollution, the leaching waste solution can be recycled through the following approach. During the leaching process, [Met][NO_3_] forms a [Met-Pb] complex with Pb^2+^ that enters the liquid phase, while unreacted [Met][NO_3_] remains active. After removing Pb^2+^ from the waste solution via “targeted removal of adsorbates to retain functional components” approach [11], sodium sulfide precipitation or electrodeposition technology, the supernatant rich in free [Met][NO_3_] can be directly reused for leaching. Before recycling, its concentration requires monitoring; if a 10–15% decrease occurs due to complexation reactions, supplementing with fresh solution to the initial concentration maintains efficiency. Additionally, Pb^2+^ separation residues (e.g., PbS) can be further recovered, forming a closed-loop system to mitigate environmental risks.

## 4. Conclusions

In this study, a series of washing experiments were conducted with AAILs as the washing agent to determine the optimal washing agent and washing conditions. When the [Met][NO_3_] washing agent was selected with a concentration of 0.8 mol/L, a washing time of 60 min, and a liquid–soil ratio of 6:1, the removal efficiency of Pb reached 85.4%. After washing, there was a notable reduction in the content of all forms of Pb, especially the carbonate-bound Pb. Furthermore, the washing process did not have any discernible impact on the soil type. The organic matter content exhibited a significant increase, whereas the pH value and mineral element content demonstrated a decline. The removal of Pb through washing with [Met][NO_3_] was identified as a heterogeneous diffusion process, with potential mechanisms including ion exchange and coordination reactions. Overall, this work provides an effective and environmentally friendly approach to the remediation of Pb-contaminated soil. In practical applications, it is necessary to consider the potential impact of washing on the environment to prevent secondary pollution. In addition, the washing process can be further optimized with the help of response surface methodology or orthogonal experimental design, and reasonable measures can be designed for the recycling of washing agents and the treatment of washing wastewater. In future research, we should conduct field trials on historically contaminated soils and carry out long-term monitoring of soil health status.

## Figures and Tables

**Table 1:** Design of soil washing experiment.

Influence Factor	Experimental Parameter
AAILs type	Washing agent concentration of 0.3 mol/L, liquid–soil ratio of 4:1, washing time of 1440 min
Washing agent concentration	Washing agent concentration of 0.1, 0.2, 0.3, 0.4, 0.5, 0.8, 1.0 mol/L, liquid–soil ratio of 4:1, washing time of 1440 min
Liquid–soil ratio	Washing agent concentration of 0.8 mol/L, liquid–soil ratio of 2:1, 3:1, 4:1, 5:1, 6:1, 7:1, 8:1, washing time of 1440 min
Washing time	Washing agent concentration of 0.8 mol/L, liquid–soil ratio of 6:1, washing time of 15, 30, 60, 120, 240, 360, 480, 720, 1440 min

**Table 2:** Tessier five-step extraction method.

Fraction	Extraction Solution	Extraction Method
F1	8 mL 1.00 mol/L MgCl_2_	Oscillation for 1 h (25 °C, 150 r/min)
F2	8 mL 1.00 mol/L CH_3_COONa (pH = 5)	Oscillation for 5 h (25 °C, 150 r/min)
F3	20 mL 0.04 mol/L NH_2_OH·HCl	Water bath heating and batch stirring for 5 h (96 ± 2 °C)
F4	3 mL 0.02 mol/L HNO_3_, 5 mL 30% H_2_O_2_ (pH = 2)	Water bath heating and batch stirring for 5 h (85 ± 2 °C)
	5 mL 30% H_2_O_2_ (pH = 2)	Water bath heating and batch stirring for 3 h (85 ± 2 °C)
	5 mL 3.20 mol/L CH_3_COONH_4_ (dilution to 20 mL with 20% HNO_3_)	Oscillation for 30 min (25 °C, 150 r/min)
F5	HNO_3_-HCl-HF	Microwave digestion

Note: F1 represents exchangeable Pb, F2 represents carbonate-bound Pb, F3 represents iron–manganese-oxide-bound Pb, F4 represents organic-bound Pb, F5 represents residual Pb.

**Table 3:** Methods or instrument for the determination of physical and chemical properties of soil.

Properties	Method/Instrument
Particle size	Laser particle size analyzer (BT-2003, Baite, Yueqing, China)
pH	pH meter (SC-619, Mettler Toledo, Zurich, Switzerland)
EC	Electrical conductivity meter (AB23EC, Ohaus, Parsippany, NJ, USA)
OM	Potassium dichromate oxidation-spectrophotometric method
CEC	Hexamminecobalt trichloride solution-spectrophotometric method
Ex-Ca, Ex-Mg	Ammonium acetate solution-atomic absorption spectrophotometry method
TN	Kjeldahl method
TP	Alkali-fusion Mo-Sb anti spectrophotometric method
TK	Alkali fusion-atomic absorption spectrophotometry
AP	Sodium hydrogen carbonate solution-Mo-Sb anti spectrophotometric method
AK	Ammonium acetate solution-atomic absorption spectrophotometry method
Ca, Mg, Mn, Fe	Microwave digestion

**Table 4:** Methods for the characterization of soil.

Characterization	Method/Instrument
Surface morphology	SEM (Tescan Mira4, Tescan, Brno, Czech Republic)
Mineral composition	XRD (Ultima IV, Rigaku, Tokyo, Japan)
Functional groups	FT-IR (Nicolet iS5, Thermo Scientific, Waltham, MA, USA)
Chemical morphology	XPS (K-Alpha, Thermo Scientific, Waltham, MA, USA)

**Table 5:** The equation of Kinetic model.

Kinetic Model	Equation
Second order kinetic model	$q_{t}=\frac{q_{e}^{2}k_{1}t}{1+k_{1}q_{e}t}$
Elovich model	$q_{t}=\frac{1}{\beta }\times ln\left(\alpha \beta t\right)$
Diffusion model	$q_{t}=k_{d}\sqrt{t}+C$

Note: *t* represents the washing time; *q_t_* and *q_e_* represent the washing amount at time *t* and at equilibrium, respectively; *k*_1_ is the second-order rate constant; α is the initial adsorption rate; *β is* the desorption constant; *k_d_* is the diffusion rate constant and *C* is a constant.

**Table 6:** Effect of soil washing on basic properties and exchange performance of soil. The [Met][NO_3_] washing agent was selected with 0.8 mol/L concentration, 60 min washing time and 6:1 liquid–soil ratio.

Characteristics		Before Washing	After Washing
Basic properties	Sand/Silt/Clay (%)	36/47/17	38/47/15
	pH	5.2 ± 0.1	2.0 ± 0.0
	EC (ms/cm)	1.9 ± 0.2	4.5 ± 0.7
	OM (g/kg)	7.2 ± 0.7	23.0 ± 0.8
Exchange performance	CEC (cmol/kg)	18.4 ± 0.1	19.4 ± 0.0
	Ex-Ca (g/kg)	4.5 ± 0.1	1.8 ± 0.2
	Ex-Mg (g/kg)	2.3 ± 0.1	2.0 ± 0.0

## Data Availability

Data is contained within the article.
